# Supplementary material for: Dual-Function Meta-Grating Based on Tunable Fano Resonance for Reflective Filter and Sensor Applications
Source: Sensors (Basel). 2023 Jul 17;23(14):6462. doi: 10.3390/s23146462 (PMC10383033; doi:10.3390/s23146462)
Supplement: Supplementary file 1 [file sensors-23-06462-s001.zip › sensors-2429400-supplementary.pdf]

sensors

Supplementary information

Dual-Function Meta-Grating Based on Tunable Fano Resonance for Reflective Filter and Sensor Applications

Feifei Liu [1,*], Haoyu Jia [1], Yuxue Chen [1], Xiaoai Luo [1], Meidong Huang [1], Meng Wang [2] and Xinping Zhang [3]

1 College Physics & Materials Science, Tianjin Normal University, Tianjin 300387, China; 2210300023@stu.tjnu.edu.cn (H.J.); 15075451840@163.com (Y.C.); luoxiaoai0304@163.com (X.L.); mdhuang@tjnu.edu.cn (M.H.)
2 School of Physical Science and Technology, Inner Mongolia University, Hohhot 010021, China; wangmeng@imu.edu.cn
3 Institute of Information Photonics Technology, College of Applied Sciences, Beijng University of Technology, Beijing 100124, China; zhangxinping@bjut.edu.cn
* Correspondence: liufeifei@tjnu.edu.cn

Figure S1 shows the electric intensity distributions of the PR grating at λ=675 nm and 725 nm, respectively. Obviously, the resonance modes at λ=675 nm and 725 nm correspond to SPP modes confined to the interface between the Au film and PR spacer layer and the interface between the Au film and SiO2 substrate, respectively. Fig. S2 shows the electric intensity distribution of the MIM meta-grating at λ=720 nm, implying that the SPP resonance is confined to the interface between Au film and SiO2 substrate. Fig. S3 shows the current and charge-density distribution of the MIM meta-grating at the resonance wavelengths of $\lambda \approx 465$ nm and 610 nm. Those results clearly show that the broadband plasmonic mode corresponds to a high-order resonance mode. Meanwhile, it has a certain radiation ability [1].

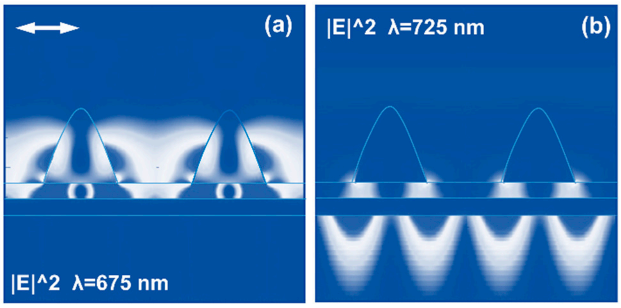

Figure S1. (a,b) Electric intensity distribution of the PR grating at λ=675 nm and 725 nm, respectively.

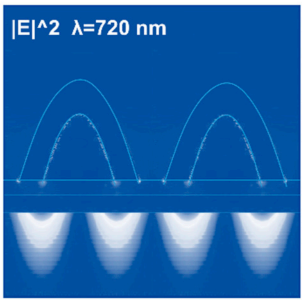

Figure S2. Electric intensity distribution of the MIM meta-grating at λ=720 nm.

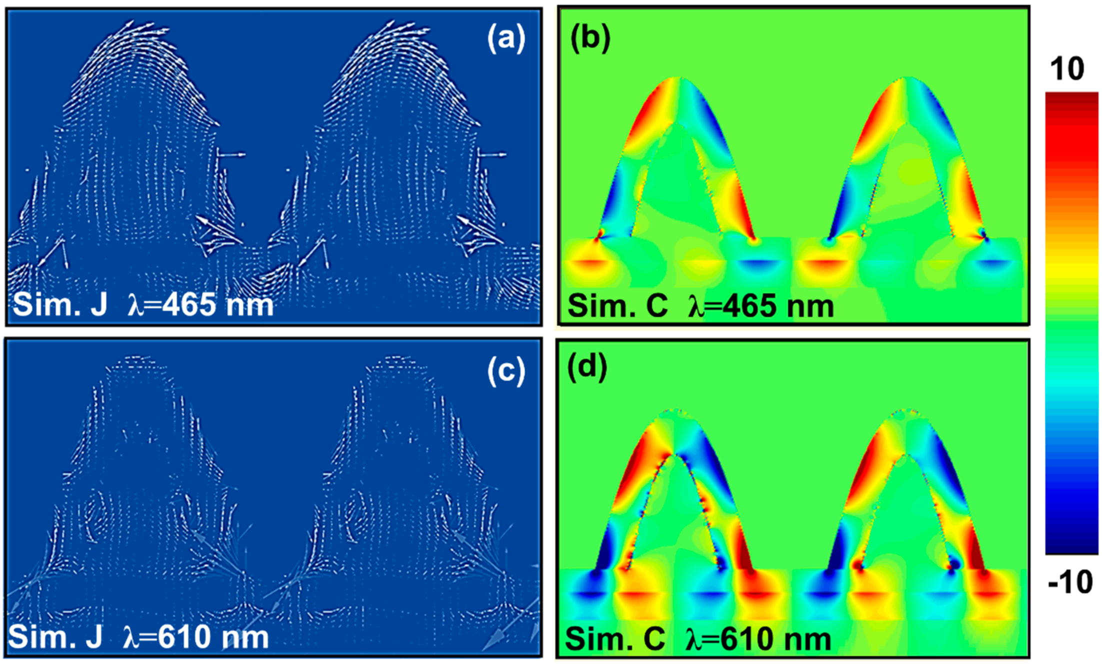

Figure S3. Current and charge-density distribution of the MIM meta-grating at the resonance wavelengths of $\lambda \approx 465$ nm and 610 nm.

Figure S4 shows the simulation configuration of the hybrid sensor detecting external environmental RI changes. As shown in Fig. S3, the incident light is located above the detected sample which is highlighted by the red box. The environmental RI in the red box is changed from 1 to 1.02 with a small step size of 0.005.

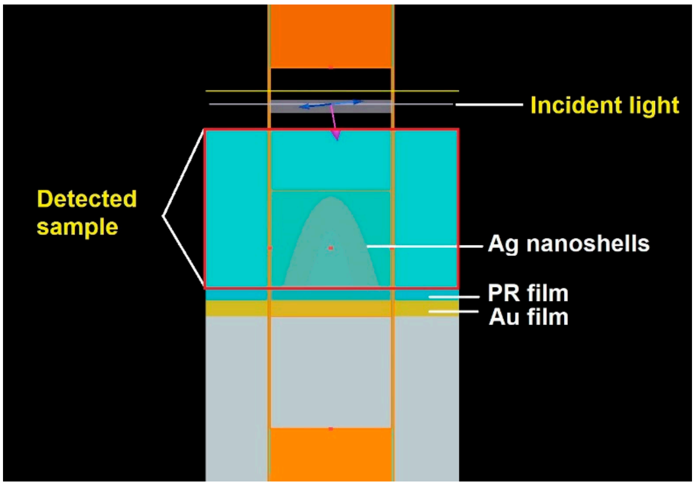

Figure S4. Simulation configuration of the hybrid sensor detecting RI changes in the external environment.

[1] Liu, F.; Zhang, X.; Yuan, Y.; Lin, J.; Wang, M.; Ma, H. Complementary dark and bright plasmonic nanocavities with controllable energy exchange for SERS sensing. *Adv. Optical. Mater.* **2020**, *8*, 2000544.
